# Associations of neurotransmitters and the gut microbiome with emotional distress in mixed type of irritable bowel syndrome

**DOI:** 10.1038/s41598-022-05756-0

**Published:** 2022-01-31

**Authors:** Zahra A. Barandouzi, Joochul Lee, Maria del Carmen Rosas, Jie Chen, Wendy A. Henderson, Angela R. Starkweather, Xiaomei S. Cong

**Affiliations:** 1 School of Nursing, University of Connecticut, 231 Glenbrook Road, Unit 4026, Storrs, CT 06269-4026 USA; 2 School of Nursing, Emory University, 1520 Clifton Rd, Atlanta, GA 30322 USA; 3 Department of Statistics, University of Connecticut, 215 Glenbrook Road, U-4120, Storrs, CT 06269-4120 USA; 4 Department of Biostatistics and Epidemiology, University of Pennsylvania, 423 Guardian Dr, Philadelphia, PA 19104 USA; 5 School of Nursing, University of Maryland, 655 W Lombard St, Baltimore, MD 21201 USA; 6 Biobehavioral Research Laboratory, School of Nursing, University of Connecticut, 231 Glenbrook Road, Unit 4026, Storrs, CT 06269-4026 USA

**Keywords:** Medical research, Outcomes research

## Abstract

Evidence highlights the comorbidity between emotional distress and irritable bowel syndrome (IBS) through the gut-brain axis. However, the underlying mechanism is largely unknown. Thus, the present study aimed to evaluate the associations among neurotransmitter levels and the gut microbiome profiles in persons with IBS and emotional distress. In this nested case-controlled study, emotional symptoms, including anxiety and depressive symptoms, were evaluated in 40 persons with IBS and 20 healthy controls (HC). Plasma neurotransmitters levels (serotonin and norepinephrine) and the gut microbiome profile of the collected fecal samples were examined. Emotional distress and microbiome profile were significantly different between IBS and HC groups. Lower but not significant neurotransmitters’ levels (serotonin and norepinephrine) were observed in the IBS group compared to the HC. A negative correlation was found between norepinephrine levels and alpha diversity (Shannon and Simpson indices) in the IBS group. Moreover, serotonin levels were positively associated with the abundance of *Proteobacteria*, and norepinephrine were positively correlated with *Bacteroidetes*, but negatively associated with *Firmicutes* phylum. The present study demonstrated alteration in the gut microbiome between persons with IBS and emotional distress compared to HC. The correlations between plasma neurotransmitters and the gut microbiome suggest that the gut microbiome may impact the regulation of neurotransmitters.

## Introduction

Irritable bowel syndrome (IBS), the most common functional gastrointestinal disorder, is characterized by chronic abdominal pain and alteration in bowel habits^1–3^. IBS affects 10–15% of adults worldwide and is associated with a broad spectrum of emotional distress comorbidities^4^. Epidemiological data report that 40–90% of persons with IBS experience some degree of anxiety and depressive symptoms in their life^5,6^. Emotional distress may influence IBS symptom perception and illness behavior in patients, contribute to poor quality of life as well as increase health care costs^7–9^. A comorbid condition may suggest a biological association between emotional distress and IBS^5^.

The relationship between IBS and emotional distress is supported by neuroimaging and psychophysiological studies^10,11^. This association may be related to the bidirectional communication between the central nervous system (CNS) and the gut neuroendocrine system known as the brain-gut axis^4,12^. Emotional distress may lead to homeostasis shifts along the brain-gut axis and alteration in the gut microbiome pattern in IBS^13^. Several studies reported similarities in the fecal microbiome of persons with IBS and persons with depression (e.g., high abundance of *Proteobacteria* and low abundance of *Bifidobacteria*), which highlights the bidirectional interaction between the brain and the gut in comorbidity between emotional distress and IBS^13,14^.

Neurotransmitter imbalance is one of the mechanisms attributed to emotional distress^15,16^. It has been postulated that insufficient levels of the monoamine neurotransmitters (e.g., serotonin, dopamine and norepinephrine) may lead to depression^17^. Serotonin, as a key neurotransmitter of the brain-gut axis, plays an important role in the pathogenesis of both emotional distress and IBS^6^. More than 90% of the body’s serotonin is synthesized in the gut^18,19^. Various bacteria such as *Streptococcus spp., Enterococcus spp., Escherichia spp., Lactobacillus plantarum, Klebsiella pneumonia,* and *Morganella morganii* have the ability to produce serotonin^20–23^. The literature also supports that other neurotransmitters such as dopamine and norepinephrine can be produced by bacteria such as *Lactobacillus, Serratia, Bacillus, Morganella* and *Klebsiella.* Interestingly, dopamine and norepinephrine can stimulate the growth of *E. coli O157: H7*^24,25^. Alteration of the gut microbiome may change the biosynthesis, release, and reuptake of neurotransmitters. The alteration may result in a disturbance in processes operating between the CNS and the enteric nervous system and may lead to emotional distress^6,26^. Described mutual interaction between the CNS and the gut highlights the role of the gut microbiome and neurotransmitters in emotional distress^27^.

Traditionally, emotional distress such as depression has been related to an imbalance in serotonin, norepinephrine, and dopamine levels. Some studies evaluated the role of serotonin in IBS, but research about the function of other involved neurotransmitters such as norepinephrine is limited^28^. Moreover, the gut microbiome as a cardinal part of the gut-brain axis has gained significant interest in the pathophysiology of both IBS and emotional distress in recent years. Thus, in the current study, we hypothesized a correlation between the gut microbiome and plasma neurotransmitters levels in IBS people with emotional distress. We aimed to investigate the difference in biomarkers (neurotransmitters levels and gut microbiome diversity) between IBS and healthy controls (HC), as well as the relationship between the gut microbiome and neurotransmitters in persons with IBS and emotional distress.

## Results

### Demographic characteristics

Demographic information of the participants, including gender, age, race, ethnicity, and marital status, is shown in Table 1. The mean age was 21 and 20 years old in the IBS and HC groups, respectively. The majority of the participants were female, White, and Non-Hispanic. The results show that the demographic characteristics were homogenous between the IBS and HC groups except in age.

**Table 1:** Demographic characteristics of the participants.

Variable	IBS (n = 40) N (%) or Mean ± SD	HC (n = 20) N (%) or Mean ± SD	*P*
**Gender**			
Female	26 (65%)	10 (50%)	.402
Male	14 (35%)	10 (50%)	
Age (years)	21.67 ± 2.22	20.10 ± 1.41	.007
**Race**			
White	31 (77%)	10 (50%)	.092
Asian	6 (15%)	6 (30%)	
African-American	3 (8%)	4 (20%)	
**Ethnicity**			
Hispanic	5 (12%)	4 (20%)	.391
Non-Hispanic	33 (83%)	15 (75%)	
Unknown	0 (0)	1 (5%)	
Not reported	2 (5%)	0 (0)	
**Marital status**			
Never married	38 (95%)	20 (100%)	.548
Married	2 (5%)	0 (0%)	

IBS, irritable bowel syndrome; HC, healthy control.

### Levels of anxiety and depressive symptoms

The mean anxiety scores were 62.85 ± 7.67 and 54.41 ± 6.12 in the IBS and HC groups, respectively. Moreover, the mean of depressive symptoms were 56.11 ± 7.30 and 48.55 ± 6.26 in the IBS and HC groups correspondingly. The results showed that the mean of anxiety and depressive symptoms were significantly higher in the IBS group compared to the HC group. While the HC anxiety and depressive symptoms were within the normal limits, the IBS group had moderate anxiety level and mild depressive symptoms. The results can be found in Table 2.

**Table 2:** Difference in anxiety and depression scores between IBS and healthy control (HC) groups.

Variable	IBS (n = 40) Mean ± SD	HC (n = 20) Mean ± SD	*p*
Anxiety	62.858 ± 7.678	54.415 ± 6.123	< .0001
Depressive symptoms	56.110 ± 7.309	48.555 ± 6.268	< .0001

IBS, irritable bowel syndrome; HC, healthy control.

### Neurotransmitter levels

The mean plasma serotonin level was 35.47 ± 27.53 ng/ml and 42.36 ± 31.94 ng/ml and the norepinephrine level was 358.66 ± 120.25 pg/ml and 395.57 ± 169.84 pg/ml in the IBS and HC groups, respectively. While plasma serotonin and norepinephrine levels were lower in the IBS group, they were not significantly different compared to the HC group. The results are shown in Table 3.

**Table 3:** Difference in plasma neurotransmitters levels between IBS and control groups.

Variable	IBS Mean ± SD		HC Mean ± SD		*p*
Plasma serotonin level (ng/ml)	35.474 ± 27.539	N = 31	42.369 ± 31.914	N = 17	.304
Plasma norepinephrine level (pg/ml)	358.660 ± 120.256	N = 30	395.579 ± 169.849	N = 14	.412

IBS, irritable bowel syndrome; HC, healthy control.

### Gut microbiome profile

#### Gut microbiome patterns

**Figure 1:**
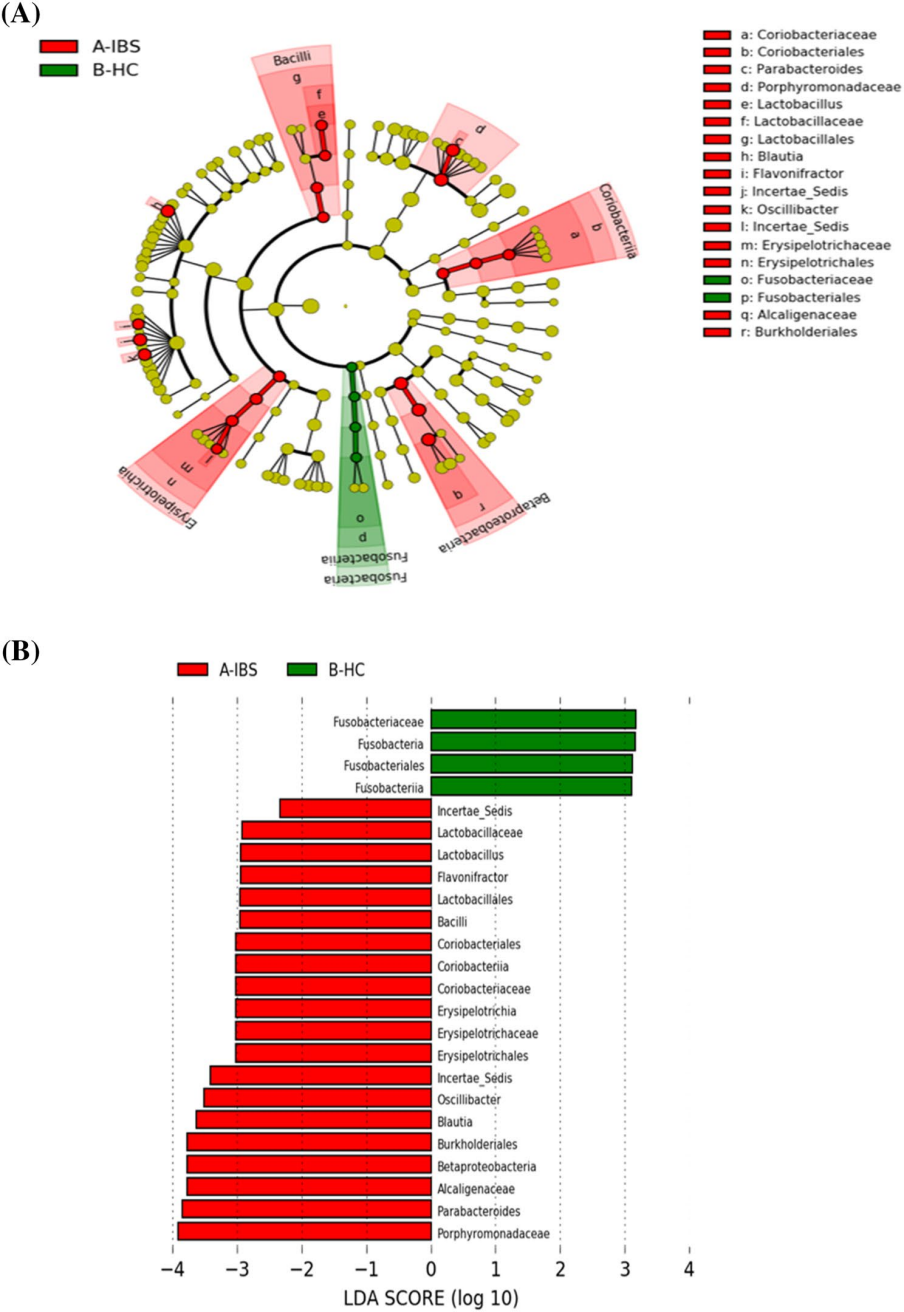
Taxonomic differences of fecal microbiome between IBS and healthy control (HC) (Green) HC taxa; (Red) taxa enriched in people with irritable bowel syndrome (IBS). (**A**) Taxonomic cladogram based on the LEfSe analysis. (**B**) IBS-enriched taxa are indicated with a positive linear discriminant analysis (LDA) score (red) and taxa enriched in HC have a negative score (green).

At the taxonomic level, among different OTUs, there was a difference in the abundance of bacteria between the IBS and HC groups. We observed high abundance of *Fusobacteriaceae, Fusobacteria, Fusobacteriales* and *Fusobacales* in the HC group and a high abundance of *Lactobacillaceae, Lactobacillus, Flavonifractor, Lactobacillales, Bacilli, Coriobacteriales, Coriobacteriia, Coriobacteriaceae, Erysipelotrichia, Erysipelotrichaceae, Erysipelotrichales, Oscillibacter, Blautia, Burkholderiales, Betaproteobacteria, Alcaligenaceae, Parabacteroides* and *Porphyromonadaceae* in perspons with IBS compared to HC (Fig. 1).

#### Gut microbiome diversity

**Figure 2:**
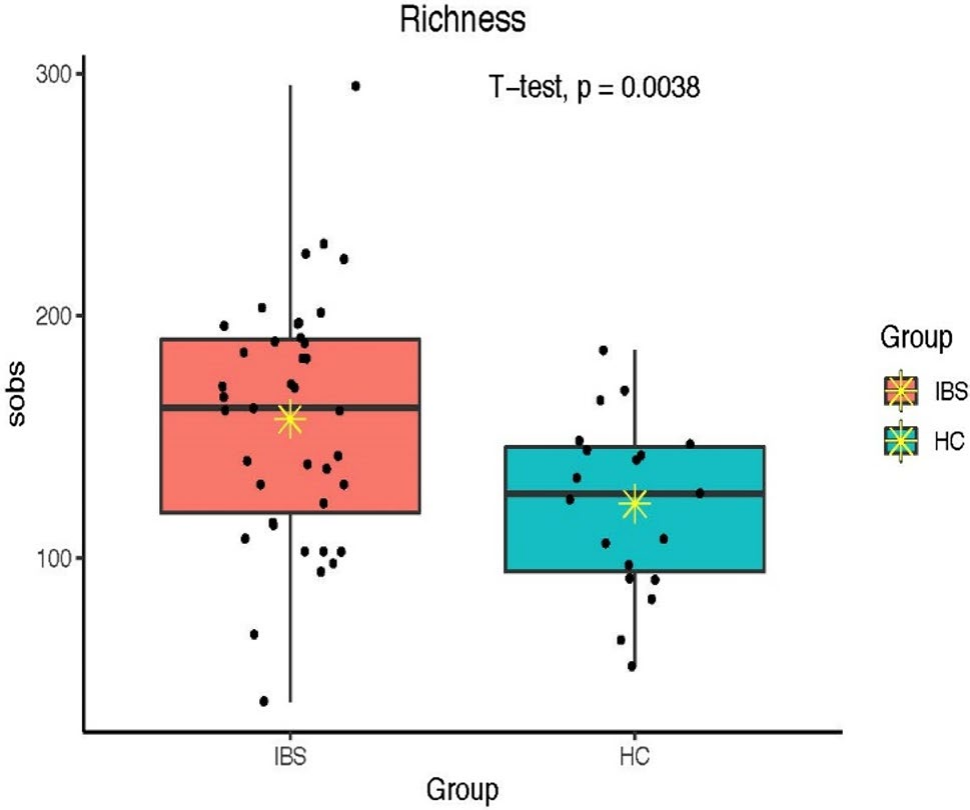
Total observed species (sobs) diversity between IBS and HC groups; * indicates mean.

**Figure 3:**
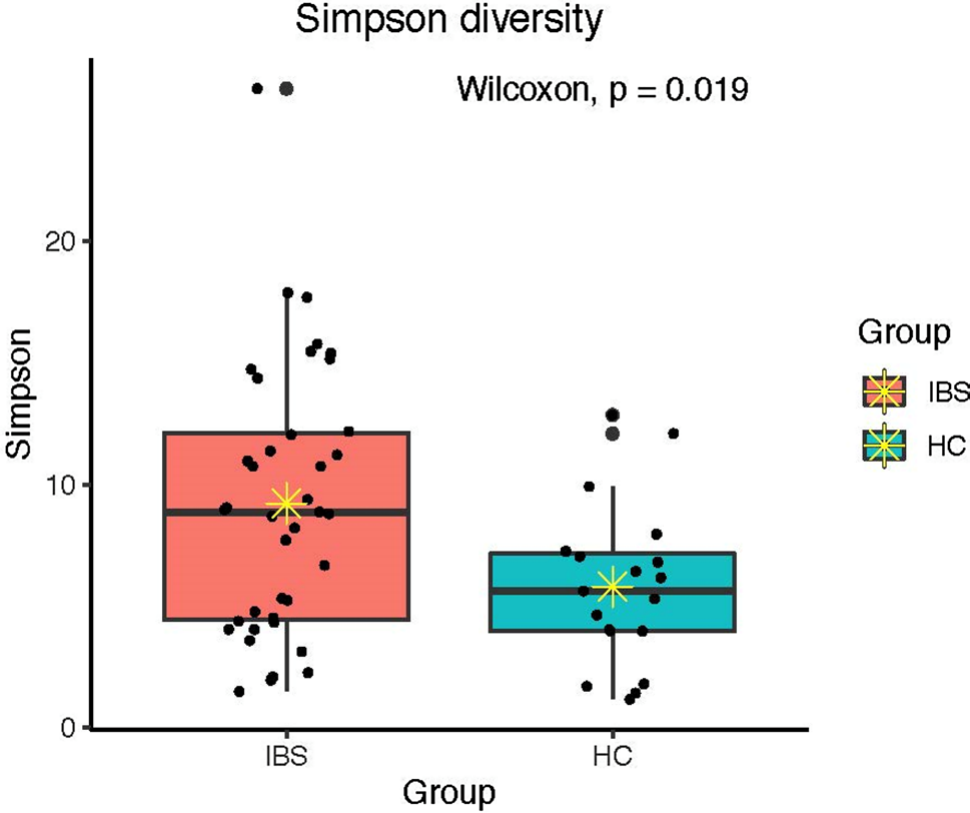
Simpson diversity between IBS and HC; * indicates mean.

**Figure 4:**
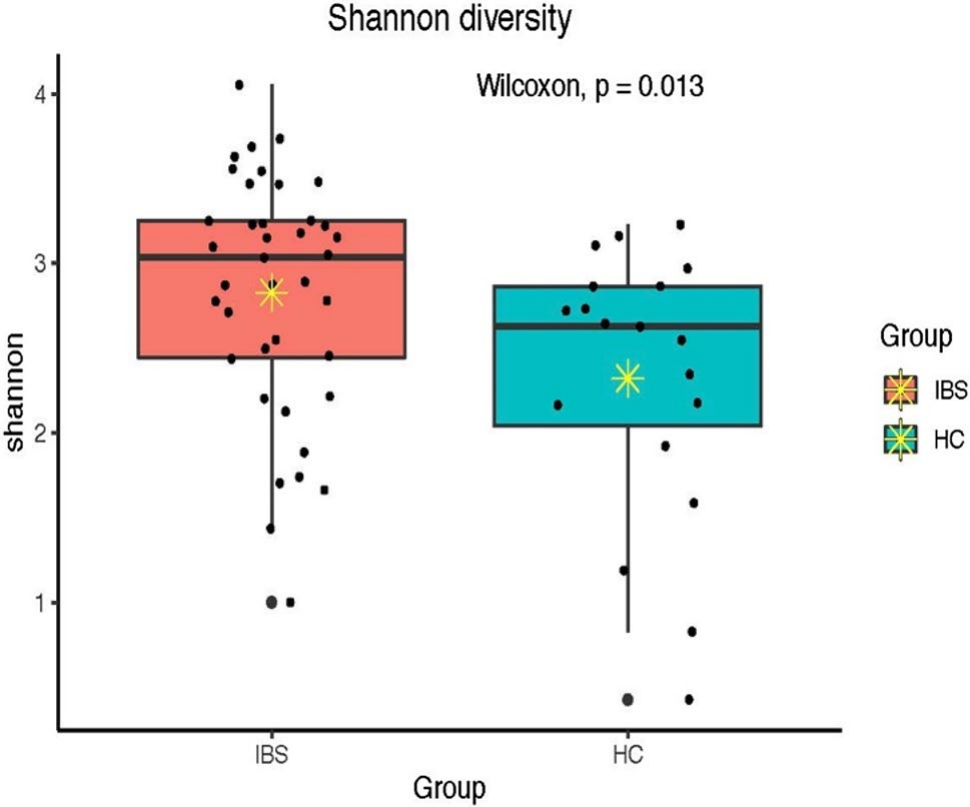
Shannon diversity between IBS and HC groups; * indicates mean.

Alpha diversity (the richness and evenness of the microbial community) was measured using three different alpha diversity indices, including total observed species (sobs), Simpson (richness and evenness of the microbial community within a sample), and Shannon (evenness of the microbial community within a sample) indices. According to these indices, there was an increased bacteria alpha diversity in the IBS group compared to the HC group (sobs (*p* = 0.003), Simpson (*p* = 0.019), and Shannon (*p* = 0.013) (Figs. 2, 3, 4).

**Figure 5:**
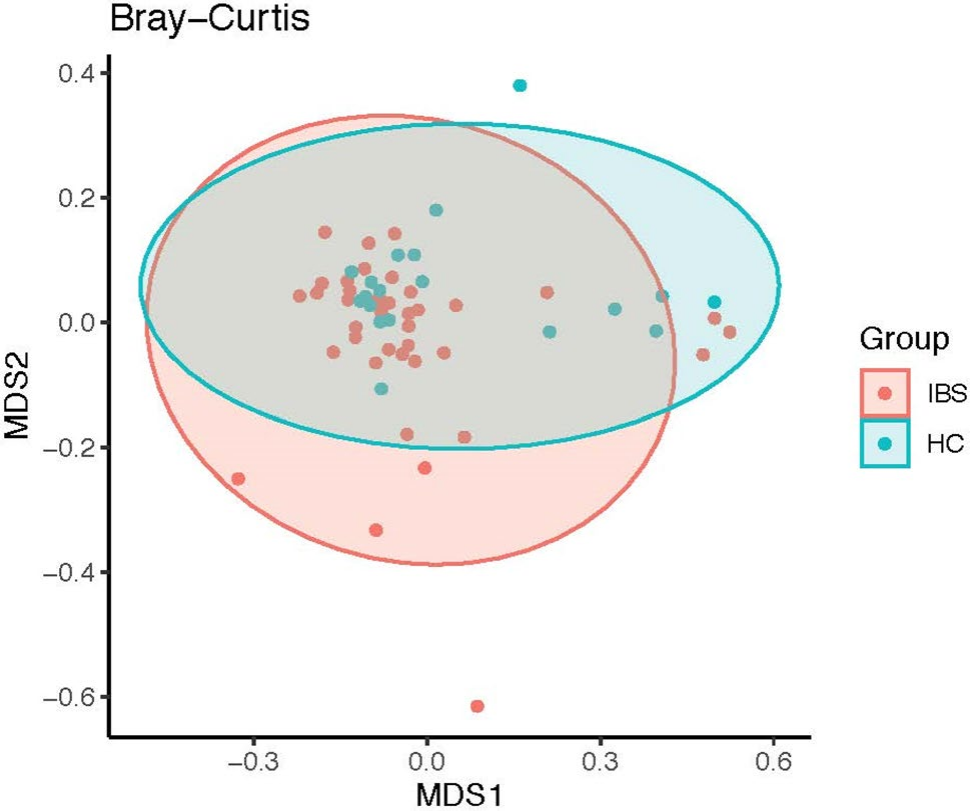
Fecal microbiome dissimilarity using Bray–Curtis distance between IBS and healthy control (HC) groups.

**Figure 6:**
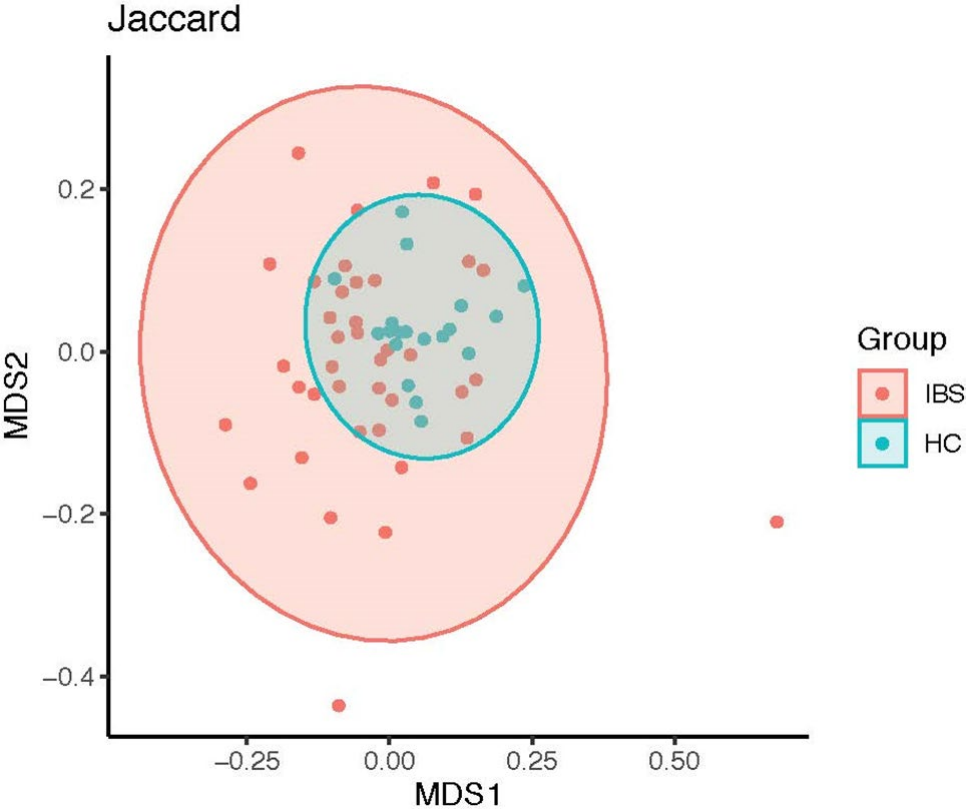
Fecal microbiome dissimilarity using Jaccard distance between IBS and healthy control (HC) groups.

Moreover, there was significant dissimilarity in beta diversity (the compositional dissimilarity among the microbiome community) using Bray–Curtis (abundance or read count data) and Jaccard (the presence/absence of species) dissimilarities between the two groups (Figs. 5, 6). The multivariate dispersions test of homogeneity assumption was satisfied for Bray–Curtis, but not for Jaccard dissimilarity.

### Association between neurotransmitter levels and the gut microbiome in IBS people

In the analysis of associations between plasma neurotransmitter levels and alpha diversity indices in the IBS group, there were no correlations between serotonin levels and alpha diversities, but norepinephrine levels were found to be negatively correlated to Shannon and Simpson indices (*p* = 0.029 and *p* = 0.015; respectively) (Table 4).

**Table 4:** Correlation between plasma neurotransmitter levels and bacterial diversity indices in IBS people.

Neurotransmitter	Diversity index	Spearman’s r	*p*
Serotonin	Sobs	.068	.717
	Shannon	.167	.374
	Simpson	.169	.369
Norepinephrine	Sobs	− .262	.168
	Shannon	− .403	.029
	Simpson	− .444	.015

In terms of associations between plasma neurotransmitter levels and bacteria phylum, we observed a significant positive correlation between serotonin levels and *Proteobacteria* (*p* = 0.021), a significant negative correlation between norepinephrine and *Firmicutes* (*p* = 0.020), and a significant positive correlation between norepinephrine and *Bacteroidetes* phylum (*p* = 0.043) (Table 5).

**Table 5:** Correlation between plasma neurotransmitter levels and bacteria phylum in IBS people.

Neurotransmitter	Phylum	Spearman’s r	*p*
Serotonin	Firmicutes	.072	.703
	Bacteroidetes	− .198	.292
	Actinobacteria	.116	.541
	Proteobacteria	.421	.021
Norepinephrine	Firmicutes	− .431	.020
	Bacteroidetes	.379	.043
	Actinobacteria	− .103	.591
	Proteobacteria	− .185	.334

## Discussion

The current study assessed the difference in the plasma neurotransmitter levels and the gut microbiome profile in persons with IBS who experienced anxiety and depressive symptoms compared to the HC. Also, we evaluated the correlation between neurotransmitter levels and the gut microbiome diversities and microbial abundance at the phylum level. Our findings revealed that gut microbiome patterns in persons with IBS who suffer from emotional distress are different from HC. Although there was no difference in neurotransmitter levels between the two groups, we observed a correlation between bacterial profile (bacterial taxa at phylum level and alpha diversity) and neurotransmitters’ levels in the IBS group.

It has been hypothesized that the imbalance in monoamine neurotransmitter levels (i.e., serotonin and norepinephrine) is a key factor in the pathophysiology of depressive symptoms and anxiety disorders^29^. Thus, high comorbidity between IBS and emotional distress, including anxiety and depressive symptoms, may be a consequence of an alteration in serotonin and norepinephrine^30,31^. Both the CNS and the enteric nervous system have the capability to produce neurotransmitters^28^. A decrease in the amount of serotonin and norepinephrine released and deterioration of neurotransmitters binding to receptors may lead to emotional distress^32^. In the current study, no significant difference was observed in either serotonin or norepinephrine levels between IBS and HC. Our results are in contrast to the findings of Zhao et al. (2014) in which IBS patients with depressive symptoms had lower plasma serotonin^33^. While serotonin has drawn a tremendous amount of attention regarding the pathogenesis of comorbidity of depressive symptoms and IBS, some studies have reported a lack of significant correlation between plasma serotonin levels and depressive symptoms in IBS patients compared to healthy controls^34–36^. Although alteration in serotonin levels has been highlighted in the comorbidity of IBS and depressive symptoms^33^, there are limited studies to investigate the role of other neurotransmitters such as norepinephrine in comorbidity between IBS and emotional distress. Thus, further research is needed to assess the role of norepinephrine in this comorbidity.

It has been postulated that the gut microbiome contributes to the pathogenesis of IBS and emotional distress, such as depressive symptoms and anxiety^18^. The gut microbiome may influence basal circuits of emotion processing via the vagal pathway from the gut^37,38^. Interoceptive stimuli may impede conscious and subconscious emotional and cognitive processing^39^. Research has shown that the dysbiosis in the gut microbiome is a shared abnormality in the pathophysiology of both IBS and depressive symptoms^26,40^. We observed higher alpha diversity in terms of total observed species, Simpson and Shannon indices in persons with IBS compared to HC. Our results contrast with the findings of Peter (2018), who reported non-significant lower alpha diversity in IBS patients with psychological distress^13^. Similarly, Hugerth (2019) found no association between bacterial diversity and depressive symptoms in IBS patients^41^. While we observed higher alpha diversity, Liu (2016) reported low alpha diversity (Shannon index) in persons with IBS and depressive symptoms^14^. Sundin (2015) also showed a negative correlation between fecal microbial diversity and anxiety and depressive symptoms in patients with IBS^42^. While pre-clinical studies have supported the hypothesis of lower alpha diversity in animal models after exposure to stress^43,44^, the results in clinical studies are contradicted and require further investigations. Regarding beta diversity, similar to our findings, Peter (2018) found that microbial beta diversity was associated with depressive symptoms^13^. Beta diversity as an indicator of taxa similarity within samples may clarify how emotional distress segregated the gut microbiome features among different subgroups of individuals that require further investigation^45^.

We also found a high abundance of several bacteria at different taxonomic levels that belong primarily to the three phyla *Bacteroidetes*, *Firmicutes,* and *Verrucomicrobia* in persons with IBS who suffer from emotional distress. Similar to our findings, elevated *Lachnospiraceae* were reported in cases of both anxiety and depressive symptoms^13^. However, studies have shown mixed results concerning the abundance of various gut microbiomes at different taxonomic levels among persons with IBS who had emotional distress, and thus, additional studies are needed^41,46^.

Recent evidence has highlighted the ability of the gut microbiome to produce neurotransmitters, which may lead to the development/maintenance of emotional distress such as anxiety and depression. While we did not observe any association between various alpha diversity indices and serotonin, there was a negative correlation between alpha diversity (Shannon and Simpson indices) and norepinephrine levels. Moreover, there was a positive association between serotonin and *Proteobacteria* phylum as well as norepinephrine and *Bacteroidetes* phylum. However, *Firmicutes* had a negative association with norepinephrine.

Research shows that more than 90% of the serotonin in the human body is synthesized by the gut^19^. For instance, *Candida, Escherichia coli, Streptococcus,* and *Enterococcus* may secrete serotonin and *Escherichia coli*, *Bacillus* and *Saccharomyces* can produce norepinephrine^47,48^. The production of neurotransmitters by various microbiota may lead to the imbalance of neurotransmitter levels and the development of pathogenic bacteria. For example, the presence of norepinephrine may stimulate the growth of *Escherichia coli*^25^. Additionally, studies show that the in vitro exposure of the *Klebsiella pneumoniae*, *Enterobacter cloacae*, *Pseudomonas aeruginosa*, *Shigella sonnei*, and *Staphylococcus aureus* to norepinephrine may lead to the growth of these pathogens^49^. So, alteration of the gut microbiome due to IBS may be attributed to the imbalance in neurotransmitters levels and subsequently emotional distress. Accumulated preclinical studies highlight the role of the gut microbiome in regulating neurotransmitters and depressive-like behavior in the animal models^50^. There is a strong need for future clinical studies to understand the biological mechanism of comorbidity of emotional distress with IBS by shedding light on the role of the gut microbiome in the production of neurotransmitters.

## Limitations

There are limitations in the study that need to be considered when interpreting the results. The small sample did not allow the categorization of persons with IBS based on the severity of the emotional distress. Therefore, the interpretation of the biomarkers based on the severity of emotional distress is limited. Moreover, a small sample size and unbalanced design might increase the likelihood of a type II error skewing the results. Therefore, caution is needed when interpreting the results of the study, which needs to be replicated in a larger sample. The majority of the participants in our study were mixed type (IBS-M) according to the self-reported symptoms. Further studies with a larger sample size by considering history of diet, IBS subtype and other IBS symptoms (e.g., pain, bowel habits) are recommended to determine the associations between neurotransmitters and the gut microbiome in persons with IBS and emotional distress.

## Conclusion

The study’s findings highlight that persons with IBS and emotional distress may be differentiated from healthy persons based on their gut microbiome profile. There were lower but not significant neurotransmitter levels in the IBS group compared to the HC. Also, we found correlation between the gut microbiome (at both diversity and operational taxonomic unit) and neurotransmitters in persons with IBS and emotional distress. Future research in a larger sample is required to correlate neurotransmitter levels with observed gut microbiome alterations. Identification of biomarkers may help advance IBS management with comorbid emotional distress.

## Methods

### Study design and subjects

The present cross-sectional comparative study is part of a parent randomized clinical trial titled “Precision Pain Self-Management in Young Adults with IBS” (NCT03332537)^51^. In the parent study, 80 persons with IBS diagnosed by a gastroenterologist were enrolled from the general community as well as two large public university campuses and two gastrointestinal (GI) clinics in the northeastern region of the United States. The participants completed emotional distress questionnaires, and their blood and fecal samples were collected at three different time points: baseline, six weeks, and 12 weeks. The data from the parent study's baseline session was utilized and 20 healthy subjects were recruited as controls in the present study.

The inclusion criteria for enrollment of people with IBS in the published protocol of the parent study were: (1) men and women 18–29 years of age, (2) with a diagnosis of IBS from a healthcare provider using the Rome IV criteria, and (3) able to read and speak in English. The exclusion criteria were: (1) having other chronic painful conditions including but not limited to fibromyalgia, chronic pelvic pain or chronic intestinal cystitis, infectious diseases (hepatitis, HIV, MRSA), celiac disease or inflammatory bowel disease, and/or diabetes mellitus, (2) serious mental health conditions (e.g., bipolar disorder, schizophrenia, mania), (3) women pregnant or post-partum within 3 months of delivery, or, (4) regular use of opioids, iron supplements, prebiotics/probiotics or antibiotics, and/or substance use. The criteria for recruitment of HC were the same as those for the IBS group, except that the HC group did not have a history of IBS or any chronic disease^52^.

### Data collection

The IBS and HC groups completed questionnaires via a Research Electronic Data Capture (REDCap) software/system. Peripheral blood samples were collected into EDTA tubes using a standardized venipuncture procedure. The collected blood samples were diluted using 2% fetal bovine serum and centrifuged at 1000×*g* for 20 min at 4 °C, and then plasma was aliquoted into eppendorf tubes and stored at − 80 °C until further analysis.

The participants were requested to collect their fecal samples at the time of enrollment in the study using a stool sample collection kit (OMNIgene•GUT, DNA Genotek Inc., Canada) per manufacturer instructions. Samples were mailed back to the research laboratory and frozen and maintained at − 80 °C until processing and 16S rRNA gene sequencing analysis.

### Outcome measures

#### Emotional distress measurement

Anxiety and depressive symptoms were assessed using the Patient-Reported Outcomes Measurement Information System (PROMIS) v1.0 questionnaires from the recommended National Institute of Nursing Research (NINR) common data elements. PROMIS instruments measure clinically important outcomes, including physical, mental, and social health. PROMIS is scored by using the Health Measures Scoring Service^53,54^.

##### Anxiety

The PROMIS questionnaire, the “Emotional Distress-Anxiety” section, which includes 6 questions, was used for measuring anxiety. Responders selected answers based on the category provided by the questionnaire (never, rarely, sometimes, often, and always). The researcher scored the responder's answers (never = 1, rarely = 2, sometimes = 3, often = 4, and always = 5). After calculating the raw score by summing responses, the total raw score was translated to a T-score which is a standardized T-score with a mean of 50 and a standard deviation of 10. According to the PROMIS measurements, a score below 55 is within the normal limit, the score of 55–60 refers to mild, and a score above 60 indicates moderate and severe anxiety symptoms^55^.

##### Depressive symptoms

For measuring depressive symptoms, the PROMIS questionnaire, “Emotional Distress-Depression” section, which includes questions with five different answer categories (never = 1, rarely = 2, sometimes = 3, often = 4, and always = 5) was used. The same approach was employed to calculate depressive symptoms according to the PROMIS measurement guidelines^55^.

#### Plasma levels of neurotransmitters measurement using ELISA

##### Serotonin

This study used the serotonin enzyme-linked immunosorbent assay (ELISA) assay kit named ADI-900-175 (Enzo Life Sciences, New York, New York) for the quantitative determination of serotonin in plasma^56^. For this purpose, the steps mentioned in the guideline of Serotonin ELISA Assay Kit, including preparation of reagents (e.g., microtiter strips, wash buffer and Equalizing Reagent), acylation of samples (20 µl) and ELISA test procedures were followed as per the manufacturer's instructions. The competitive Serotonin ELISA Assay kit uses the microtiter plate format, in which serotonin is bound to the solid phase of the plate. Antirabbit/peroxidase detects the antibody bound to the solid phase serotonin. The level of serotonin in the sample is inversely proportional to the amount of antibody bound to the solid phase serotonin. The sensitivity of the kit was 0.293 ng/ml and the range of detection was 0.49–500 ng/ml. The samples were run in duplicate, per batch and the mean inter- and intra-assay coefficients of variation for control samples was ≤ 20%.

##### Norepinephrine

For the quantitative determination of norepinephrine in plasma, the norepinephrine ELISA assay kit named KA1891 (Abnova, Taipei, Taiwan) was used^57^. For this purpose, 300 µl of plasma was processed according to the guideline of Norepinephrine ELISA Assay Kit, including preparation of reagents (e.g., wash buffer and enzyme mix), preparation of samples, and ELISA test procedure per manufacture’s directions. The kit's sensitivity was 5 pg/ml and the range of detection was 0–32 pg/ml. The samples were run in duplicate, per batch and the mean inter- and intra-assay coefficients of variation for control samples was ≤ 20%.

#### Microbiome sequencing and analysis

The fecal samples DNA extraction, sequencing, and analysis were carried out at the University of Connecticut, Center of Microbial Analysis, Resources, and Services (UConn MARS). Bacterial DNA was extracted from 0.25 g of stool sample using the MoBio PowerSoil isolation kit (Mo Bio Laboratories, Inc, CA, USA) in line with the manufacturer’s instruction for the Eppendorf epMotion 5076 Vac liquid handling robot^58^. Then the V4 region of the 16S rRNA genes of the microbial community were sequenced using the MiSeq Series Illumina platform.

The sequences produced by PCR were normalized based on the concentration of DNA in the 350–400 bp region and pooled using the QIAgility liquid handling robot (Qiagen). Then, the pooled PCR was purified using GeneRead Size Selection kit (Qiagen, Hilden, Germany, cat. Cat. ID: 180514) and paired-end sequencing (2 × 250) was performed on an Illumina MiSeq system.

The raw sequence data were processed by Mothur 1.42.3 Pipeline^59^ following the analysis pipeline of Miseq SOP (http://www.mothur.org/wiki/MiSeq_SOP) and the UConn MARS mothur batch file (https://github.com/krmaas/bioinformatics/blob/master/mothur.bash). Paired-end sequences were combined into contigs and aligned against to the SILVA 119 V4 16 S rRNA gene reference alignment database^60^. Operational taxonomic units (OTUs) were determined at a 97% identify, and then performing de novo OTU clustering on reads that failed to cluster to a reference. Taxonomic annotation was also determined by the SILVA 16S rRNA reference dataset. Sample were removed if the yielded reads were less than 10,000. For the microbiome analysis, the Mothur software^59^ was used. Alpha diversity, including Simpson, Shannon, and total observed species (sobs) indices, were used to evaluate the complexity of the whole microbial community. Beta diversity represented by Bray–Curtis and Jaccard was used to indicate the inter-subjects’ dissimilarity in the bacterial communities.

### Statistical analysis

For data analysis, the SPSS (statistical package for social sciences) version 26 and R version 4.0.2 were used. The demographic characteristics were presented with mean and standard deviation for continuous variables and frequency and percentage for continuous variables. The statistical approaches to assess the difference in emotional distress, and neurotransmitter levels between IBS and HC groups were Independent Sample T-test and Mann–Whitney. For analysis of microbiota composition, the linear discriminant analysis effect size (LEfSe) method was performed^61^. For comparing alpha diversity between the IBS and HC groups, a Wilcoxon rank-sum test was used. Also, based on Bray–Curtis and Jaccard dissimilarity, non-metric multidimensional scaling (NMDS) ordination for beta diversity was performed and multivariate dispersions (PERMDIST) test was conducted to examine the assumption of homogeneity. Spearman rank correlation was used to examine the association between neurotransmitter levels and alpha diversity indices (Simpson, Shannon and sobs) as well as bacterial phylum in the IBS group.

### Ethics approval and consent to participate

The study was approved by the Institutional Review Board of the University of Connecticut and was conducted in accordance with the ethical guidelines of the Declaration of Helsinki. All the participants provided written informed consent.

## Data Availability

The datasets generated analyzed in the current study are available from the NCBI dBGaP (https://submit.ncbi.nlm.nih.gov/subs/sra/SUB8914789/).
